# Exploring barriers to and facilitators of adaptation in family caregivers of stroke survivors: a qualitative study

**DOI:** 10.1186/s12913-026-14883-7

**Published:** 2026-06-05

**Authors:** Mahnaz Moghadari Koosha, Azim Azizi, Alireza Torabi, Masoud Khodaveisi, Efat Sadeghian

**Affiliations:** 1 School of Nursing and Midwifery, Hamadan University of Medical Sciences, Hamadan, Iran; 2 Chronic Diseases (Home Care) Research Center, Institute of Cancer, Hamadan University of Medical Sciences, Hamadan, Iran; 3 Department of Medical Surgical Nursing, School of Nursing and Midwifery, Hamadan University of Medical Sciences, Hamadan, Iran; 4 Clinical Research Development Unit of Besat Hospital, Hamadan University of Medical Sciences, Hamadan, Iran; 5 Department of Community Health Nursing, School of Nursing and Midwifery, Hamadan University of Medical Sciences, Hamadan, Iran; 6 Department of Nursing, School of Nursing and Midwifery, Hamadan University of Medical Sciences, Hamadan, Iran

**Keywords:** Adaptation, Family Caregivers, Stroke, Qualitative study, Resilience

## Abstract

**Background:**

The sudden transition into caregiving following a stroke presents significant challenges for family members, profoundly affecting their well-being and capacity to adapt. A comprehensive understanding of the barriers and facilitators involved is essential for the development of effective support mechanisms, particularly in resource-limited contexts. This study investigates these factors among family caregivers of stroke survivors in Iran.

**Methods:**

This qualitative study employed conventional content analysis. Twelve participants were selected through purposive sampling with maximum variation. Data obtained from in-depth, semi-structured interviews were analyzed utilizing Graneheim and Lundman’s framework.

**Results:**

Twelve family caregivers (9 women, 3 men; mean age 45 years) participated in the study. Analysis generated three integrative themes. First, caregivers described a multidimensional erosion of well-being, including physical exhaustion, psychological identity disruption, financial strain, and loss of personal space. Second, adaptation was mediated through culturally grounded meaning-making, kinship support, knowledge acquisition, and episodic healthcare assistance. Third, adaptation emerged as a dynamic balancing process in which caregivers managed cumulative burdens through active coping, identity reformation, and conditional growth within structurally limited support systems.

**Conclusion:**

Caregiver adaptation following stroke reflects a dynamic balance between cumulative burden and contextually mediated reconstruction. Rather than signaling resolution of hardship, adaptation involves the conditional reorganization of identity, relationships, and everyday practices within structural constraints. Recognizing caregivers’ simultaneous vulnerability and resourcefulness offers a culturally grounded framework to inform responsive, context-sensitive caregiver support policies and interventions.

**Supplementary Information:**

The online version contains supplementary material available at 10.1186/s12913-026-14883-7.

## Background

Stroke is a prevalent neurological disorder and represents a significant global health challenge [1]. It is the second leading cause of death worldwide and ranks third in numerous regions, following cardiovascular diseases and cancer, with an annual incidence ranging from 2.7 to 4.7 cases per 1,000 individuals [2, 3]. This condition frequently leads to substantial cognitive, memory, and physical impairments, resulting in severe disability [4, 5]. More than 75% of stroke survivors experience persistent deficits, necessitating long-term family support following discharge [6, 7].

Families encounter abrupt physical and emotional stressors when undertaking caregiving responsibilities for stroke survivors, which require significant lifestyle adjustments to ensure effective care [8, 9]. Caregivers often prioritize the needs of the stroke survivors over their own health, frequently receiving inadequate support from healthcare systems or governmental resources [5]. In comparison to caregivers of other neurological conditions, those caring for stroke survivors report elevated levels of depression and anxiety [10]. The impact of stroke on family dynamics can diminish caregiver well-being and may lead to difficulties in adaptation [8].

Adaptation encompasses a range of strategies employed to manage stressful situations, whether these are perceived or actual threats [11]. Effective adaptation is crucial for patients and families as they navigate chronic changes [4, 12–14], ultimately influencing recovery outcomes [15]. Nursing interventions, including education and counseling, play a vital role in providing support to families [2]. However, there is a paucity of evidence regarding the barriers and facilitators to caregiver adaptation, which hinders the development of evidence-based strategies for post-stroke health [16]. Existing studies identify emotional distress, emotion-focused coping, social support, role changes, and social participation as critical factors influencing caregiver experiences [17], yet they often neglect to consider cultural variations.

Stroke constitutes a significant and unpredictable life-altering event, affecting not only the survivor but also their family, who must suddenly undertake complex and enduring caregiving responsibilities [18]. While the general challenges encountered by caregivers—such as emotional distress, role strain, and unmet informational needs—are well-documented in the literature, a critical knowledge gap persists [19, 20]. Specifically, there is a deficiency in comprehensive, contextual understanding of how adaptation is negotiated within non-Western, resource-limited settings where formal support systems are scarce and reliance on informal, culturally embedded resources is essential [20].

Existing studies, predominantly conducted in Western contexts, underscore factors such as social support and education but frequently neglect the unique interplay of cultural, spiritual, and familial capital that mediates resilience within specific sociocultural environments [21]. For example, research within the Iranian context identifies the utilization of religiosity as a coping strategy [19], yet the underlying mechanisms through which spiritual frameworks, such as Thwab (divine reward) and Hurmat-e Valedayn (sanctity of parents), fundamentally shape caregiving identity, meaning-making, and perseverance remain inadequately explored. Moreover, the significant tension between these robust informal support systems and the widespread “structural abandonment” by formal healthcare systems—an endemic characteristic in many low- and middle-income countries—has not been sufficiently theorized [20].

This study seeks to address this critical gap by employing a qualitative approach to elucidate the complex systemic barriers and facilitators of adaptation among family caregivers of stroke survivors in Iran. It aims to provide a nuanced, contextual understanding of how adaptation is dynamically constructed within a complex interplay between substantial burdens and informal, culturally-rooted resources. Our findings are positioned to advance the Roy Adaptation Model by empirically elaborating on the “contextual stimuli” (particularly cultural and systemic factors) that shape caregiver responses. This research will inform the development of culturally sensitive, family-centered support programs that leverage existing cultural strengths while advocating for essential structural reforms in post-discharge care.

## Methods

### Design

This study was conducted using a qualitative conventional content analysis approach according to the method proposed by Graneheim and Lundman (2004). This approach was chosen to explore and describe the barriers to and facilitators of adaptation among family caregivers of stroke survivors, allowing for the extraction of manifest and latent meanings from their lived experiences [22].

### Reflexivity statement

The research team actively engaged in reflexive practice to mitigate the influence of our professional backgrounds on the findings. The primary researcher, a neurologist nurse, maintained a reflective journal throughout the study. Early in data collection, she recognized a tendency to interpret caregiver statements through a clinical lens. Through journaling and self-correction, she consciously employed open-ended questions and active listening to prioritize caregivers’ narratives, a practice of “bracketing” her clinical assumptions to allow their lived experiences to emerge authentically.

Data analysis involved a multidisciplinary team, crucial for managing bias. Regular peer debriefing sessions facilitated open discussion of reflective journal entries and coding frameworks. Team members challenged interpretations and assumptions, exploring alternative meanings. For example, a proposed code for “emotional burden” was refined through peer discussion to differentiate between grief, anxiety, and daily care strain, ensuring interpretations were anchored in participants’ words. This iterative process of reflection, bracketing, and peer discussion significantly shaped our coding and interpretation decisions, ensuring the final themes accurately represented the data rather than researcher-imposed constructs.

### Study setting and participant selection

Participants in this study consisted of 12 primary family caregivers of stroke survivors who were selected through purposive sampling with maximum variation in terms of age, gender, relationship to the patient, and duration of caregiving (Table 1). All participants were currently caregiving at the time of interview. The study setting included the neurology wards and outpatient clinics of Besat hospital in Hamadan, Iran. To ensure a calm environment and maintain confidentiality, all interviews were conducted in a quiet and private room within the hospital. Inclusion criteria were as follows: participants had to serve as the primary informal caregiver (spouse, adult child, or parent) of a stroke survivor; co-reside with the patient or provide ≥ 4 h of direct caregiving per day; having at least 6 months of caregiving experience; care for a survivor with moderate to severe functional disability (Modified Rankin Scale score 3–5); communicate fluently in Persian and participate in in-depth interviews; have no pre-existing major psychiatric diagnosis prior to the index stroke (confirmed via psychiatric evaluation or structured screening); have no physical disability that would impair caregiving; and have experienced no major stressful life event (e.g., bereavement, divorce, significant financial loss) within six months preceding enrolment. Exclusion criteria included voluntary withdrawal of consent or expressed unwillingness to continue participation at any point.

Recruitment procedure: To ensure methodological transparency and minimize selection bias, a specific recruitment protocol was followed. Potential participants were initially identified through patient records and consultations with ward nurses and neurologists. However, to ensure that clinicians did not act as “gatekeepers” who might influence participation, they only acted as facilitators by identifying eligible families. The primary researcher (MNK) then personally approached the caregivers to explain the study’s purpose, ensure voluntariness, and obtain informed consent. This direct communication allowed for a neutral recruitment process independent of the clinical care team.

**Table 1:** Demographic characteristics of the participants

Participant Code	Age (Age group)	Gender	Marital Status	Relationship with the patient	Time since stroke onset (months)	Duration of Interview (minutes)
1	40–45	Female	Married	Patient’s Spouse	6	70
2	15–20	Female	Single	Patient’s Daughter	12	45
3	30–35	Male	Single	Patient’s Son	13	40
4	40–45	Female	Married	Patient’s Daughter	8	45
5	40–45	Female	Married	Patient’s Spouse	7	40
6	45–50	Female	Single	Patient’s Daughter	9	35
7	70–75	Female	Married	Patient’s Spouse	18	30
8	50–55	Female	Married	Patient’s Spouse	8	40
9	40–45	Female	Single	Patient’s Daughter	12	40
10	50–55	Female	Married	Patient’s Daughter	6	45
11	30–35	Male	Single	Patient’s Son	15	35
12	60–65	Male	Married	Patient’s Spouse	7	40

### Data collection

Data were collected through semi-structured, in-depth, face-to-face interviews. The interviews began with a broad, open-ended question: “Could you please share your experience of caring for your patient since the stroke occurred?” Probing questions such as “What has helped you cope?” or “What made this process difficult for you?” were used to gain deeper insights. Each interview lasted between 30 and 70 min. All interviews were audio-recorded with the participants’ permission and transcribed verbatim.

Data saturation was achieved when no new codes or conceptual insights emerged from the interviews, and the identified categories reached a state of stability. In this study, code redundancy was observed by the 10th interview. To ensure that the categories were robust and that no further nuances remained undiscovered, two additional interviews were conducted (total *n* = 12), which confirmed that the data had reached full saturation and the categories were well-defined.

### Data analysis

The data were analyzed utilizing the method established by Graneheim and Lundman for conventional qualitative content analysis. This process entailed verbatim transcription of interviews followed by the identification and condensation of meaning units. These units were subsequently coded, and through comparative analysis, the codes were organized into subcategories and categories based on identified similarities and differences. Ultimately, by interpreting the underlying meanings across these categories, overarching themes were developed to encapsulate the latent content of the data. This iterative process involved continuous comparison between the data and the evolving analysis to ensure that the findings remained anchored in the participants’ experiences [22]. Table 2 provides a concrete example of this progression from a raw quotation to the final theme.

**Table 2:** Example of the progression from meaning unit to theme

Analytical Step	Output
1. Quotation	(*“I spend my entire pension on medicines and diapers. Nothing is left to run the household. It’s like I’m falling into a bottomless pit.“*)
2. Meaning Unit	Expenditure of all income on medical supplies, leaving no resources for basic household needs, resulting in a sensation of financial helplessness.
3. Code	Catastrophic depletion of financial resources
4. subcategory	The Direct, Back-Breaking Costs of Treatment
5. Category	Economic Strain and Structural Vulnerability
6. Theme	The Multidimensional Erosion of Caregiver Well-Being

### Rigor (Trustworthiness)

Lincoln and Guba’s criteria—credibility, dependability, confirmability, and transferability—were applied to ensure trustworthiness [23].

To enhance the credibility of the data, prolonged engagement with the subject was implemented. Additionally, four family caregivers of stroke patients corroborated our findings through their experiences and statements (member checking). To enhance dependability, a preliminary literature review was undertaken to reduce potential researcher bias. An external auditor (a faculty reviewer) examined the interviews, coding process, interpretations, and findings throughout the study to ensure methodological rigor. Confirmability was supported by maintaining a comprehensive audit trail documenting all methodological decisions and analytic procedures, which was reviewed by the external auditor. In addition, peer debriefing and expert checking were conducted. The preliminary coding framework and categories were evaluated by a panel of two neurologists and two nurses specializing in stroke care to ensure alignment between the extracted themes and the clinical realities of caregiving. Transferability was facilitated through maximum variation sampling [24].

### Ethics considerations

This study, which is part of a nursing Ph.D. dissertation, received approval from the Ethics Committee and Research Council of Technology at Hamadan University of Medical Sciences (IR.UMSHA.REC.1401.949). All participants were informed of the study’s objectives and procedures, and their participation was entirely voluntary, with the option to withdraw at any stage. Following each interview, participants were presented with a gift as a token of gratitude for their involvement in the research.

## Results

In the present study, a total of 12 interviews were conducted with 12 patient caregivers. Among the participants, 9 were female, and 3 were male. Of these, 7 participants were married, and 5 were single. The distribution of family caregivers in relation to the patients was as follows: 5 were spouses of the patients, 5 were daughters, and 2 were sons. The average age of the participants was 45 years (Table 1). The analysis yielded three integrative themes, reflecting a dynamic process in which caregivers negotiate overwhelming burdens through culturally mediated resources and adaptive reconstruction of self and family life (Table 3).

**Table 3:** Thematic framework of barriers to and facilitators of adaptation in family caregivers of stroke survivors

Theme	Category
1. The Multidimensional Erosion of Caregiver Well-Being (Core Barriers)	1.1 Physical Exhaustion and Self-Neglect
	1.2 Psychological Disruption and Identity Fracture
	1.3 Economic Strain and Structural Vulnerability
	1.4 Loss of Personal Space and Environmental Intrusion
2. Informal Resources and Meaning Systems (Facilitators of Adaptation)	2.1 Cultural-Spiritual Meaning-Making as Emotional Regulation
	2.2 Kinship Networks and Relational Reorganization
	2.3 Knowledge and Competence as Instrumental Empowerment
	2.4 The Healthcare System: Episodic Support within Structural Abandonment
3. Adaptive Reconstruction: Managing a Dynamic Balance	3.1 Active Coping and Strategic Management
	3.2 Reconstructed Identity and Emergent Strength
	3.3 Conditional Growth within Persistent Vulnerability

## The multidimensional erosion of caregiver well-being (Core Barriers)

Rather than discrete categories of burden, participants described a cumulative erosion of physical integrity, psychological coherence, economic security, and personal space. These stressors operated synergistically, progressively destabilizing caregivers’ sense of self and their capacity to continue caregiving.

### Physical exhaustion and self-neglect

Caregivers experienced relentless bodily strain from lifting, transferring, and assisting with ADLs.

My hands and back are in constant pain from lifting him… (P1).

This physical depletion was inseparable from progressive self-neglect. Sleep fragmentation, irregular meals, and abandonment of personal medical needs formed a cycle of deterioration. Caregivers described their bodies as resources progressively exhausted.

I haven’t had more than two consecutive hours of sleep in months… (P8).

### Psychological disruption and identity fracture

Emotional distress and identity disruption emerged as intertwined phenomena. Caregivers simultaneously mourned the altered condition of their loved one and the significant change in their own former identity. Anxiety, grief, secondary trauma from behavioral changes, and loss of relational reciprocity coalesced into a profound undermining of their self-concept.

My father sits silently… I miss the father he used to be. (P6)

I am no longer a counselor, a mother, a partner. I am only ‘my mother’s caregiver. (P4)

Thus, psychological suffering was not limited to emotional symptoms; it involved a profound disruption to their sense of being, where the caregiving role overshadowed their previous identities.

### Economic strain and structural vulnerability

Direct treatment costs and indirect income loss produced financial insecurity.

My entire salary disappears into medicines… (P12).

I had to close my shop… (P3).

Financial strain intensified psychological stress and restricted access to formal support, reinforcing dependence on informal networks.

### Loss of personal space and environmental intrusion

The transformation of the home into a quasi-clinical environment represented a blurring of boundaries between caregiving and personal life. Participants described not merely spatial change, but the disappearance of sanc.

tuary and privacy, deepening emotional exhaustion.

My home is no longer my sanctuary… (P5).

## Informal resources and meaning systems (facilitators of adaptation)

In the absence of robust formal support, adaptation was primarily mediated through culturally embedded belief systems and relational resources. These did not eliminate burden but reframed and redistributed it.

### Cultural-spiritual meaning-making as emotional regulation

Spiritual beliefs functioned simultaneously as moral obligation and emotional coping mechanism. Concepts such as Thwab (divine reward) and Hurmat-e Valedayn (sanctity of parents) structured caregiving as sacred duty, while prayer and trust in God enabled emotional regulation and adaptation.

This is thwab… it transforms suffering into patience. (P9)

Providing care was never a choice… (P11).

Meaning-making was therefore not only theological but psychological: it prevented resentment, legitimized sacrifice, and stabilized identity.

### Kinship networks and relational reorganization

Family support functioned along a continuum from fragmentation to cohesion. When effectively mobilized, kinship networks redistributed labor and created emotional containment. In some cases, crisis catalyzed strengthened bonds and collaborative identity as a caregiving unit.

My sisters and I established a schedule… (P6).

This disaster brought us closer… (P4).

Conversely:

My siblings have disappeared… (P8).

Thus, relational resources could either buffer burden or amplify isolation, depending on cohesion and equity.

### Knowledge and competence as instrumental empowerment

Practical instruction from healthcare professionals enhanced self-efficacy.

*“That five-minute lesson was more valuable than all the sympathy…”* (P10).

Conversely, informational voids intensified anxiety:

No one told me aggression was common… (P7).

Knowledge transformed caregiving from chaotic reaction to structured management, marking a critical shift from helplessness to agency.

### The healthcare system: episodic support within structural abandonment

Participants described rare humanistic encounters:

When the doctor sat beside me… (P5).

However, systemic neglect dominated:

After discharge, you are cast adrift… (P9).

Caregivers experienced the system not as a continuous support structure but as episodic and unreliable, compelling reliance on private moral and familial infrastructures.

## Adaptive reconstruction: managing a dynamic balance

Adaptation did not emerge as a linear outcome but as an ongoing process of managing the dynamic balance between cumulative burden and available resources. This process involved cognitive reframing, behavioral reorganization, and reconstitution of identity.

### Active coping and strategic management

Caregivers employed integrated coping repertoires that combined problem-solving (researching and organizing medications) with meaning-based reframing. Rather than distinct categories, these strategies functioned interactively to maintain dynamic balance.

I became a researcher overnight… (P10)

This belief is my anchor… (P9)

### Reconstructed identity and emergent strength

Over time, some caregivers described the emergence of a reconstructed identity characterized by resilience, assertiveness, and expanded competence. While the former self was disrupted, a new self— based on responsibility and strength—gradually formed.

I had to learn to be a lioness… (P2)

This reconstruction did not negate suffering but reflected adaptive integration within constrained conditions.

### Conditional growth within persistent vulnerability

For some participants, adversity generated relational closeness and personal growth. However, such growth remained conditional upon the continued availability of informal support and internal meaning systems. In the absence of these, vulnerability predominated.

This disaster brought my brothers and me closer than we have ever been. We found a unity in this duty I never knew was possible. Before this, we were just siblings; now, we are a team (P4).

### Integrative interpretation

Overall, caregiver adaptation was not simply the presence or absence of coping strategies, but an active process of managing a dynamic balance within a structurally unsupported environment. Barriers (physical depletion, psychological fracture, financial strain, spatial loss) continuously pressured caregivers toward breakdown, while facilitators (cultural meaning systems, kinship cohesion, and knowledge acquisition) served to counterbalance the pressures. Adaptive processes involved strategically navigating these forces to maintain equilibrium, ultimately resulting either in fragile resilience or cumulative exhaustion.

## Discussion

This qualitative study elucidates the barriers to and facilitators of adaptation among family caregivers of stroke survivors in Iran, revealing three overarching themes: (1) The Multidimensional Erosion of Caregiver Well-Being (Core Barriers), (2) Informal Resources and Meaning Systems (Facilitators of Adaptation), and (3) Adaptive Reconstruction: Managing a Dynamic Balance. The findings indicate that caregiver adaptation is neither linear nor uniformly progressive; rather, it constituted an active process of managing a dynamic balance between cumulative depletion and context‑mediated resources [15–17].

Consistent with Theme 1, ‘The Multidimensional Erosion of Caregiver Well-Being,’ caregivers in this study experienced substantial physical exhaustion, psychological strain, financial pressure, and disruption of personal boundaries. However, unlike studies that frame these experiences primarily as “care burden,” the present findings conceptualize them as a multidimensional erosion of well-being, reflecting progressive destabilization across physiological, emotional, relational, and existential domains. Instead of interpreting these pressures solely as individual burden, the findings highlight how structural constraints and caregiving demands interact to gradually diminish caregivers’ adaptive capacity [20, 21, 25].

Psychological distress in this study was closely intertwined with identity transformation. Caregivers described mourning the former identity of their loved ones while simultaneously reconstructing their own roles and sense of self. This aligns with prior qualitative research documenting identity shifts among long-term caregivers [14, 18]. The integration of emotional upheaval and identity disruption under the broader construct of psychological adaptation clarifies that distress and self-redefinition are not separate phenomena but interdependent processes [14, 17]. Theoretically, this resonates with the self-concept and role-function modes of the Roy Adaptation Model [26], although the findings suggest that adaptation unfolds as a fluctuating process rather than a stable end-state. Rather than confirming theoretical universality, the findings suggest that Roy’s framework is context-sensitive and mediated by sociocultural realities [26].

The second Theme, ‘Informal Resources and Meaning Systems,’ demonstrated that adaptation was not solely determined by burden. Kinship networks, experiential learning, and culturally grounded meaning-making practices functioned as buffering mechanisms. Similar to findings in collectivist cultural settings [27], family cohesion and shared responsibility contributed to emotional adaptation. However, support was uneven and sometimes fragile, reinforcing that relational resources are dynamic rather than inherently protective.

Cultural-spiritual meaning systems were particularly salient. Caregivers described caregiving as morally meaningful, spiritually framed, and embedded within familial obligation. Previous studies have identified spirituality as an emotion-focused coping strategy [14, 28], yet the present findings extend this understanding by showing that cultural-spiritual capital operates not only as coping but also as a stabilizing narrative that legitimizes sacrifice and sustains identity coherence under strain [27, 28]. In the Iranian sociocultural context, caregiving is strongly shaped by norms of filial piety and collective duty, which construct family members—particularly adult children and spouses—as morally responsible for sustaining care. These obligations are further intensified by gendered expectations that position women as primary caregivers, reflecting broader societal norms that naturalize their caregiving labor. Importantly, such resources mitigated distress but did not eliminate structural pressures [20, 21].

The third Theme, ‘Adaptive Reconstruction: Managing a Dynamic Balance,’ captures the synthesis of erosion and resource mobilization. Adaptation emerged when caregivers were able to reorganize daily routines, recalibrate expectations, and manage relational boundaries. This dynamic aligns partially with principles of Family Resilience Theory [29], particularly regarding relational reorganization during crisis. However, adaptation in this context appeared conditional and resource-dependent rather than uniformly transformative. The concept of Relational Autonomy further illuminates how caregiving decisions were embedded within moral interdependence rather than autonomous individual choice [30, 31]. Rather than fusing these frameworks, the findings suggest that each illuminates distinct dimensions of adaptation—physiological regulation (Roy) [26], relational restructuring (Family Resilience) [29], and morally embedded agency (Relational Autonomy) [30–32].

Building on these theoretical and empirical insights, several implications for clinical practice and policy emerge. From a practice perspective, these findings underscore the necessity of structured post-discharge caregiver preparation and sustained follow-up services. Caregivers require skills-based training, psychological screening, and accessible consultation pathways. Recognizing caregivers as secondary care recipients, rather than merely extensions of the patient, may reduce long-term erosion of well-being [6, 33, 34]. Strengthening transitional care coordination could mitigate cumulative strain identified in the erosion theme. At the same time, culturally responsive interventions should acknowledge spiritual meaning systems without assuming that cultural obligation neutralizes caregiver vulnerability [27, 28].

This study has limitations. The findings reflect experiences within a specific sociocultural context and may not be fully transferable to other settings. Most participants were women, potentially shaping the gendered construction of obligation and identity. Additionally, the cross-sectional qualitative design captures experiences at a particular moment rather than longitudinal adaptation trajectories. Despite the implementation of confidentiality safeguards and non‑directive, open‑ended questioning to encourage authentic responses, the possibility of social desirability bias remains. Caregivers may have selectively framed their accounts to project heightened resilience, responsibility, or emotional control, potentially shaping the interpretation of the study’s findings. Future research may benefit from longitudinal and cross-cultural comparisons to refine understanding of how erosion and reconstruction evolve over time.

## Conclusion

In conclusion, caregiver adaptation following stroke is best understood as a dynamic equilibrium between multidimensional erosion and contextually mediated reconstruction. Adaptation does not signify the resolution of hardship but the conditional reorganization of identity, relationships, and daily practices within structural constraints. By situating caregiver experiences within both relational and structural realities, this study contributes a balanced conceptualization of vulnerability and resourcefulness that may inform culturally attuned caregiver-support policies.

## Supplementary Information

Below is the link to the electronic supplementary material.

Supplementary Material 1

## Data Availability

All the data generated during this study are included in this published article.
