# Supplementary material for: Exploring barriers to and facilitators of adaptation in family caregivers of stroke survivors: a qualitative study
Source: BMC Health Serv Res. 2026 Jun 5;26:1101. doi: 10.1186/s12913-026-14883-7 (PMC13471527; doi:10.1186/s12913-026-14883-7)
Supplement: Supplementary file 1 [file 12913_2026_14883_MOESM1_ESM.docx]

**Interview Guide**

- Could you please share your experience of caring for your patient since the stroke occurred?

- Can you give examples of situations that were very difficult for you?

- Does anyone help you with her care?

- What has helped you cope?

- What made this process difficult for you?

- How has her illness affected you?

- How did you cope with this situation?

- How did you deal with psychological issues?

- Can you tell us about your experience in the hospital? Did the nurses and doctors help you for adaptation?

- How was their training? What did they do to help you accept this situation?

- Can you compare your situation at the beginning of the illness to now?

Do you have anything else you would like to say?
